# Limiting life-sustaining treatment for very old ICU patients: cultural challenges and diverse practices

**DOI:** 10.1186/s13613-023-01189-8

**Published:** 2023-10-27

**Authors:** Michael Beil, Peter Vernon van Heerden, Gavin M. Joynt, Stephen Lapinsky, Hans Flaatten, Bertrand Guidet, Dylan de Lange, Susannah Leaver, Christian Jung, Daniel Neves Forte, Du Bin, Muhammed Elhadi, Wojciech Szczeklik, Sigal Sviri

**Affiliations:** 1 Department of Medical Intensive Care, Hadassah Medical Center and Faculty of Medicine, Hebrew University of Jerusalem, Jerusalem, Israel; 2 General Intensive Care, Hadassah Medical Center and Faculty of Medicine, Hebrew University of Jerusalem, Jerusalem, Israel; 3 Department of Anaesthesia and Intensive Care, Faculty of Medicine, The Chinese University of Hong Kong, Hong Kong SAR, China; 4 Intensive Care Unit, Mount Sinai Hospital, University of Toronto, Toronto, Canada; 5 Department of Research and Development, Haukeland University Hospital, Bergen, Norway; 6 INSERM, Institut Pierre Louis d’Epidémiologie et de Santé Publique, AP-HP, Hôpital Saint Antoine, Service MIR, Sorbonne Université, Paris, France; 7 Department of Intensive Care Medicine, University Medical Center, University Utrecht, Utrecht, The Netherlands; 8 General Intensive Care, St George’s University Hospitals NHS Foundation Trust, London, UK; 9 Department of Cardiology, Pulmonology and Vascular Medicine, Faculty of Medicine, Heinrich-Heine-University, Düsseldorf, Germany; 10 Hospital das Clínicas da Faculdade de Medicina da Universidade de São Paulo, São Paulo, Brazil; 11 Medical Intensive Care Unit, Peking Union Medical College Hospital, Beijing, China; 12 Faculty of Medicine, University of Tripoli, Tripoli, Libya; 13 Center for Intensive Care and Perioperative Medicine, Jagiellonian University Medical College, Ul. Wrocławska 1-3, 30 – 901 Kraków, Poland

**Keywords:** Critical care, End of life, Guidelines, Intensive care unit, Withdrawing, Withholding

## Abstract

**Background:**

Decisions about life-sustaining therapy (LST) in the intensive care unit (ICU) depend on predictions of survival as well as the expected functional capacity and self-perceived quality of life after discharge, especially in very old patients. However, prognostication for individual patients in this cohort is hampered by substantial uncertainty which can lead to a large variability of opinions and, eventually, decisions about LST. Moreover, decision-making processes are often embedded in a framework of ethical and legal recommendations which may vary between countries resulting in divergent management strategies.

**Methods:**

Based on a vignette scenario of a multi-morbid 87-year-old patient, this article illustrates the spectrum of opinions about LST among intensivsts with a special interest in very old patients, from ten countries/regions, representing diverse cultures and healthcare systems.

**Results:**

This survey of expert opinions and national recommendations demonstrates shared principles in the management of very old ICU patients. Some guidelines also acknowledge cultural differences between population groups. Although consensus with families should be sought, shared decision-making is not formally required or practised in all countries.

**Conclusions:**

This article shows similarities and differences in the decision-making for LST in very old ICU patients and recommends strategies to deal with prognostic uncertainty. Conflicts should be anticipated in situations where stakeholders have different cultural beliefs. There is a need for more collaborative research and training in this field.

## Introduction

Decisions of when to initiate and continue life-sustaining therapy (LST) for very old patients (more than 80 years of age) in the intensive care unit (ICU) depend on the expected outcome in terms of survival as well as the achievable quality of life (QoL) after discharge. Contrary to historical perceptions, the benefit of admission to the ICU for the survival of these very old patients can be greater than in younger cohorts [1]. In fact, the oldest old patients without major co-morbidities appear to have an excellent survival rate in the ICU [2]. Although data on QoL in very old ICU survivors are scarce, a recent study in COVID-19 patients aged 70 years and older suggested that half of the survivors had no severe decline in QoL at 3 months after admission to ICU [3].

Regardless of these statistical findings, there is substantial uncertainty when predicting survival or future QoL for the individual patient [4]. Given this background, opposing opinions can arise about the benefit of burdensome interventions for patients who might be considered at the end of their life or for whom a persistent deterioration of QoL is deemed as unacceptable. This problem occurs at all major decisional milestones ranging from admission to the ICU to initiation of end-of-life (EoL) care [5] and may lead to divergent approaches to limitations of LST including communication with patients and families [6, 7]. Opinions of healthcare professionals vary with geographical locations and culture as well as personal preferences and experience [8–11]. This situation may be profoundly problematic [12]. Its wider implications have been illustrated by the controversies about ICU triage during the COVID-19 pandemic causing conflicts between professional opinions and fundamental legal principles in several countries [11, 13–19].

In the past decades, there have been numerous articles about the ethical and legal challenges involved in the withholding (WH) or withdrawing (WD) of LST. Several problems already identified in an expert report on this topic in 2004 [20], such as the consequences of prognostic uncertainty and the importance of shared decision-making, are still central to the debate today. Moreover, the rising number of very old ICU patients with complex co-morbidities has further complicated intensive care [21, 22]. In the absence of robust evidence to guide the management of these patients, decisions about the proportionality of intensive care are frequently derived from personal experience and expert opinions.

The goal of this article is to demonstrate the persistent variability of opinions about limiting LST between different countries/regions (Australia, Brazil, Canada, China, England, Hong Kong SAR, Israel, Libya, Norway, Poland). Ten intensivists present their views on the management of very old patients and discuss relevant laws and guidelines in their country/region based on a vignette scenario [23]. Although this article cannot cover all viewpoints worldwide, we have selected these countries/regions across six continents to obtain a broad spectrum of opinions embedded in different cultures and diverse healthcare systems to illustrate the diversity of approaches and identify areas for further research.

## Case scenario

The vignette scenario provides incremental challenges for decision-making in the ICU (Box 1). A list of questions (Box 2) guides the presentation of opinions about intensive care in this case.

**Box 1:** Vignette scenario

*Background:* this is an 87-year-old patient, living independently but with new mobility issues (hip osteoarthritis) requiring a stick, clinical frailty scale (CFS) 4, and without cognitive impairment. She has no opinion about limiting medical care *Past medical history:* chronic obstructive pulmonary disease (COPD) with 1–2 exacerbations per year, non-ST segment myocardial infarction (NSTEMI) 6 years ago (recent echocardiography: left ventricular ejection fraction 40%, intermediate probability of pulmonary hypertension), hip osteoarthritis, osteoporosis with vertebral fractures *Present complaints:* coughing and progressive shortness of breath for 2 days (throat swab: metapneumovirus), new palpitations, new leg oedema, confused for 12 h *Treatment in the emergency room:* non-invasive ventilation (NIV) for hypoxia and hypercapnia, failed due to confusion *Treatment in the ICU:* invasive ventilation, increasing vasopressor requirements, not fluid-responsive, inotropes added, persistent atrial fibrillation acute kidney injury (AKI) and renal replacement therapy (RRT) from day 2 upper gastro-intestinal haemorrhage on day 4 embolic stroke with hemiparesis on day 6 ventilator associated pneumonia (VAP) on day 8

**Box 2:** Questions

Which additional disease- or context-related factors would have influenced the decision to admit this patient to the ICU? What pragmatic ways would you use to deal with prognostic uncertainty? How do you assess which treatment path is in the best interest of the patient? When and how would you involve the family/surrogates in the decision-making about continuation of LST? How do you deal with divergent opinions? Would you seek the opinion of colleagues from other specialties, such as geriatric medicine or palliative care? What would be triggers to limit LST by withholding or withdrawing treatment? What is the legal framework for these decisions? Are there ethics guidelines in your country/region?	

## Expert opinions

Intensivists from ten different countries were selected due to their special interest and academic expertise in very old ICU patients. They were identified through the network of the VIP study group [24]. The experts argue their case about decisions for the patient described in Box 1 and present the underlying framework of national laws and guidelines (Table 1). Crucial components of the decision-making process are summarised in Table 2.

**Table 1:** Policies and guidelines for withholding (WH) or withdrawing (WD) life-sustaining therapy (LST) in the ICU

Country/region	Options to limit LST	Decision-maker for incapacitated patients	Guidelines and legal framework
Australia	WH and WD	Intensivist, family, friends	Regional (state) legislation, Statement on withholding and withdrawing treatment by CICM and ANZICS (2021) [25]: The potential benefits of treatment must be weighed against the burden based on probability rather than certainty There is no obligation to initiate therapy known to be ineffective, nor to continue therapy that has become ineffective When any or all aspects of active treatment are to be withheld or withdrawn, consideration should be given to comfort care
Brazil	WH and WD	Intensivist in agreement with family, often requiring consensus of the referring physician and/or specialty	Resolution of the Brazilian Federal Council of Medicine (2006) [28] In the terminal phase of serious and incurable diseases, the physician is allowed to limit or suspend procedures and treatments that prolong the patient's life, guaranteeing the necessary care to alleviate the symptoms […], respecting the patient will or her legal representative” Resolution from Sao Paulo State Medicine Council (2022) [29]: Futile treatments should not be performed, even at the request of the patient or his/her relative Regarding potentially inappropriate treatments, consensus between the healthcare team and the patient and his/her relatives is necessary for decisions
Canada	WH and WD	Intensivists for WH, substitute decision-maker (SDM) for WD	Provincial legislation, Position paper by the Canadian Critical Care Society (2017), Statement by the College of Physicians and Surgeons of Ontario (2023) [34, 35]: Physicians are not required to perform CPR when it falls outside of the usual standard of care WD of LST requires consent by patients or SDM. This is not required for WH, but SDM need to be informed WD of LST is always associated with an appropriate increase in symptomatic treatment
China	WH, rarely WD	Intensivist and family	None
England	WH and WD	Intensivist	Guidelines by the General Medical Council (GMC) for treatment and care towards the end of life (2022) [37]: There is no absolute obligation to prolong life irrespective of the consequences for the patient and his/her view It is the treating physician's responsibility to make decisions in the patient's best interest Consultation with family / carers and members of the healthcare team should be made before reaching a decision about LST
Hong Kong	WH and WD	Intensivist with participation of families	Guidelines of the Medical Council (2022) and Hospital Authority (2020) [27, 28]: Futility can be viewed in the strict sense of physiologic futility which is assessed by the health care team. In other clinical situations where futility is considered, the decision involves balancing the burdens and benefits of the treatment towards the patient. As this involves QoL considerations and can be value-laden, the decision-making is a consensus-building process between the health care team and the patient and family. In Chinese culture, the concept of self may be different from the Western concept and is more of a relational one. The role of the family in decision-making may also be more important than that of Western societies Doctors are not obliged to comply with requests that make inequitable demands on resources available to them Symptom control, comfort care and emotional support to the patient should always be offered
Israel	WH, WD only of intermittent therapies	Senior intensivist after consultations with other stakeholders (family, caregivers, legal guardians appointed by the court)	The Dying Patient Act (2006) [42]: A "dying patient" is defined as one who is not expected to survive for more than six months despite medical therapy The law tries to balance between the sanctity of life as a critical value in Jewish law and the need to respect patients' autonomy. Patients' wishes should be respected as long as they do not include active euthanasia or active shortening of the dying process This law permits WH of LST if they are futile or the patient refuses them. The law differentiates between continuous life-sustaining treatment which must not be withdrawn and intermittent treatment which may
Libya	(controversial)	Intensivist after consultation with family	None
Norway	WH and WD	Intensivist	Guideline by the Norwegian Directorate of Health (2013) [43]: Decisions concerning life-prolonging treatment must be informed by what in the patient’s best interests, and by the patient’s own wishes. The attending physician has a duty to ensure that the benefits of LST outweigh the adverse effects on the patient from the treatment or the disease. If the basis for a decision is uncertain, treatment must be initiated until its benefit has been ascertained No one can be required to administer life-prolonging treatment that is futile When life-prolonging treatment is withdrawn, palliative care should be continued
Poland	WH and WD (WD rarely used for invasive ventilation)	Intensivist consensus, often after consultations with other specialties	Guidelines by the Polish Society of Anaesthesiology and Intensive Care (2014) and Polish Society of Internal Medicine (2023) [47, 48]: There is no obligation to initiate therapy known to be ineffective (futile), nor to continue therapy that has become ineffective. The medical assessment of previous treatment and medical history should be done by a committee consisting of medical professionals. Evaluation of assumed patient's best interest is the most important value Therapeutic options and end-of-life treatment is discussed with the family which, however, cannot decide for the patient When the decision to limit LST is taken, the palliative care interventions need to be continued

**Table 2:** Components of decision-making for very old ICU patients

Country/region	Key parameters of decision-making (in addition to acute and chronic conditions)	How to deal with prognostic uncertainty	Involvement of geriatric medicine/palliative care	Trigger to consider limiting LST in the presented scenario
Australia	Expected functional outcome	Treatment trial in- or outside ICU	Rarely	AKI
Brazil	Patient's and family's values	Time-limited trial in ICU	Rarely	AKI requiring dialysis
Canada	Patient's and family's values	Time-limited trial in ICU	Palliative care	AKI, stroke
China	Family's wishes	ICU trial, discussion with patient's legal representative	Palliative care	Lack of appropriate response to treatment
England	Baseline function, expected functional outcome	ICU trial	Palliative care, geriatric medicine (rarely)	AKI
Hong Kong	Family's wishes	Time-limited trial in ICU	Rarely	High vasopressor doses, AKI, stroke
Israel	Expected functional outcome, family's wishes	ICU trial	Geriatric medicine (rarely)	Increased suffering, decreased likelihood of acceptable functional outcome
Libya	Patient's and family's values, functional independence, social support	Discussions with family, colleagues and other healthcare staff	Physicians with experience in geriatric medicine or palliative care	Individual (depending on patient's values)
Norway	Patient's values	Treatment trial in- or outside ICU	Rarely	AKI
Poland	Expected functional outcome, burden of treatment	Time-limited trial in ICU	Rarely	Stroke

*AKI* acute kidney injury

### Australia

This elderly woman presents with a clinical picture fairly typical for her age. I believe that in most ICUs in Australia this patient would not have been excluded based solely on her frailty state, age and co-morbidities. The main criteria for consideration for ICU admission would be reversibility of the presenting condition, i.e. the ability of the patient to overcome the presenting complaint and be discharged in a condition similar to her baseline. Therefore, if the prediction was that she would not survive or would survive, but at a much lower level of quality and function, then ICU admission may not have been considered (after discussion with the patient and the family).

Two pragmatic ways to deal with an uncertain outcome in this scenario would be to either assess the patient’s response to maximum non-invasive therapy outside of the ICU, but guided by an intensivist. This may include non-invasive ventilation, intravenous fluid, antibiotics, chest physiotherapy and oral vasopressor agents. Close monitoring of vital signs and urine output would also be required. If the patient showed a positive response to this therapy over a trial period of some hours then ICU admission would be considered. If the patient deteriorated then this may be taken as a sign that the patient is non-responsive to treatment and has poor physiological reserve and should therefore not be admitted to the ICU. New-onset delirium would be considered part of the presenting complaint and would be managed with pharmacological and non-pharmacological measures.

Another approach would be to admit the patient to the ICU in the first instance for a trial of therapy, after careful discussion with patient and surrogates. If, as in this case, the patient not only did not respond to therapy, but in fact developed multiple-organ failure, then treatment could be limited and the patient allowed to die.

Regarding the best treatment options for this patient, I would still be guided by the concept of reversibility and response to treatment. If the goal is to allow the patient to overcome the presenting complaint then almost any treatment option should be considered. If it becomes more and more clear that the condition is not reversible, then I would very carefully exclude treatments which cause suffering or are burdensome for the patient. I would discuss this outlook with the patient and surrogates, as well as gain the impression of colleagues before excluding specific treatments, such as renal replacement therapy. If there are divergent opinions either with colleagues or family members then I would allow for more time and repeated discussion in order to clarify the position and view the patient’s response to treatment.

Triggers for WH or WD of LST would be based on the patient’s clinical condition, the patient’s response to therapy, developing complications and the wishes and opinions of the patient (if known) and her family. My own opinion is that the dice was cast on day 2 when acute kidney injury intervened. Not achieving the goal of reversing the presenting complaint would be the main trigger to WH or WD of LST. The onset of multiple-organ failure would not only impede the reversal of the exacerbation of COPD she presented with, but would almost certainly guarantee her death despite our best efforts.

The framework for WH and WD of LST in Australia is defined in statute, i.e. the laws of the Australian states and territories, as well as by the two main professional bodies governing the speciality of intensive care (Table 1). The College of Intensive Care Medicine of Australia and New Zealand and the Australian and New Zealand Intensive Care Society have developed a joint statement after long-discussion and with the consensus of intensive care stakeholders [25] which is widely accepted as a basis for practice in EoL situations (Table 1). In general terms, decisions about LST are made by intensivists, with occasional referral to palliative care physicians. Geriatricians are rarely consulted, unless they are the initial referring doctors. This may be either due to a paucity of geriatricians or a current lack of coordination with this specialty.

### Brazil

This is a typical case that would be admitted in our ICU. This unit is part of a public hospital and supports the Emergency Room by admitting patients who still need a consensual plan and goal of treatment. Decisions about ICU admissions vary across Brazil due to its continental size and inequality which creates challenges, especially when dealing with frail and very old patients [26]. Physicians' education on EoL issues also plays a role for the variability of these decisions [27]. Despite constraints on resources in many public hospitals, however, WH or WD of LST is not common. There are no laws regulating WH or WD of LST in Brazil. Recommendations are provided by resolutions of the Brazilian Federal Council of Medicine [28] and the Sao Paulo State Medicine Council [29] (Table 1).

On the day of admission of this patient, we would focus on LST, treating potentially reversible causes while trying to set up a family meeting as soon as possible. In the first meeting, we would focus on trust building and tuning into emotions, while collecting additional information and sharing what we know and what we are uncertain about in this case. We would propose a time-limited trial, usually for 3–5 days, to reduce uncertainty and help families build trust and cope with the situation. We implemented a four-step framework to guide decisions for such patients in our ICU [30]. The first step focuses on assessing the severity of the disease by the SAP3 or SOFA score to obtain a probabilistic prognosis. For instance, at day 2, the SOFA score could be 15 (cardiovascular 3, respiratory 4, renal 4, neurologic 3, haematologic 1, gastro-intestinal 0) implying that almost 9 of 10 similar patients would die in the hospital. This patient’s frailty, her co-morbidities and multiple complications during ICU admission would further diminish her chances of survival. The few patients, who might survive, will likely suffer from functional and cognitive decline.

The second step in our framework focuses on the patient’s values. We do not ask the relatives directly what they want or which values are important to them. Instead, we would foster substitutive judgment by asking, for instance, 'how was the patient dealing with her diseases before admission'. These discussions about values are highly loaded with emotions. Family members will not share personal information if they do not trust the ICU team. Learning how to first build trust, and then how to connect with these strong emotions to help families deliberate after they calm down, have been significant lessons from the COVID-19 pandemic [31]. Demands for futile or inappropriate treatment are especially common during the initial phase in the ICU. We have learned that these requests are often expressions of despair and cold responses using probabilities turn the interaction into a battlefield. Facing such demands, I would patiently listen to the family without counterargument, I would just acknowledge the emotion by showing commitment and that I am not contradicting their hope. Then, when the family oscillates towards fear, I would again acknowledge how distressing such a situation is and then try to bring the patient’s values into the centre of our conversation.

As this patient deteriorates and the uncertainty about a negative outcome decreases, we would schedule additional family meetings. As the chance of what this patient deemed reasonable for her QoL fades, we would focus our attention on symptom control and refrain from causing any additional suffering, for example, by dialysis or CPR. In our experience, this happens often as a process, starting with WH of LST. If the patient fails to improve or further deteriorates in the following days, we would suggest to move to comfort care, allowing a natural death. We would start with WD of vasopressors, and then, mechanical ventilation. Not uncommonly, some patients get better with less aggressive care and are transferred to hospice.

### Canada

This case report describes a woman of 87 years old with an acute, potentially reversible condition, quite appropriate for critical care interventions. Although frailty is associated with increased mortality related to ICU care [32], a score of 4 is at the mild end of the spectrum. Her other co-morbidities are not contraindications to aggressive care. However, her course rapidly shows evidence of increasing severity of illness and decreasing likelihood of a good outcome. The need for inotropes alone is not a major concern, but the development of AKI markedly decreases the likelihood of surviving the hospital stay. The embolic stroke will add significant post-ICU co-morbidity and potentially reduced QoL, and the VAP adds days on the ventilator, further impacting overall outcome.

In Canada, the diverse population results in a wide variety of cultural and religious beliefs regarding EoL care. While many patients and families may opt for a more symptom-based management approach, many will ask for aggressive ICU measures. As the healthcare system is publicly funded, there is no financial pressure on families in making these decisions. The approach of most intensivists will be to review the risks and impacts of ICU care with the patient and family. With the longitudinal data generated locally [33] we know that ICU survivors are at risk of significant adverse physical and mental effects. Patients and families are usually informed of these risks, and that embarking on an ICU course is not a simple decision. As in the case presented, we know that additional complications in the ICU add to mortality risk, and a suggestion is often for a time-limited trial of ICU care. Palliative care physicians support the ICU team in communication of these issues with family members and provide ongoing support for those who leave the ICU for EoL care.

In the presented case, if the patient is no longer competent to make healthcare decisions, we would meet with the patients substitute decision-maker(s). A substitute decision-maker (SDM) is the person legally authorised to make treatment decisions on behalf of an incapable patient. Criteria and hierarchy for who can be a SDM is set out in legislation, e.g. power of attorney for personal care for spouse, child, parent and sibling. In the situation where no SDM is available, the Office of the Public Guardian and Trustee takes on this role. The SDM is made aware that they are required by legislation to make decisions based on the previously expressed wishes of the patient, and if there are no wishes, then based on the patient’s best interests. SDM are not allowed to make decisions based on their own desires. The patient's new, poor prognostic factors post-ICU admission would be presented, likely with a recommendation to focus entirely on comfort, including the WD of LST. WD of LST is always associated with an appropriate increase in symptomatic treatment and can be presented as a change in overall management goals from preserving life at all costs to a goal of optimising comfort.

The Canadian Critical Care Society has put out a position paper on WH and WD of LST, which covers the legal and ethical principles [34] (Table 1). WH and WD of LST may not be considered different, but families and healthcare staff may be more uncomfortable with the active act of WD. Canadian provinces may vary in their legal approach, but all would accept WD of LST if consented to by the patient or SDM. Although some treatments may be withheld without consent, WD of LST would require consent by SDMs as emphasised by a legal precedent in the province of Ontario. The decision to restrict or limit CPR (Do Not Resuscitate (DNR) orders) can create conflict between family and healthcare providers. Families may be concerned that other medical care may be neglected. Provincial authorities differ in their approach to the need for consent for DNR orders, but patients and/or SDMs need to be informed of this decision. It is generally accepted that physicians are not required to perform CPR when it falls outside of the usual standard of care. The College of Physicians and Surgeons of Ontario has a recent statement on 'Decision-making for end-of-life care' which confirms that physicians must obtain consent before WD of life-support [35]. However, consent is not required to withhold resuscitative measures, but SDMs do need to be informed of this decision.

### China

Understanding the patient’s wishes about QoL will be very helpful for the decision about admission to the ICU. In China, living wills are still uncommon and the best interest might have different meanings for the patient vs. the family, sometimes even within the family. It is not uncommon for the family to take on the responsibility of decision-making, sometimes against the patient’s wishes. Therefore, discussion with the family is very important, to understand the value of family vs. patients. Most families ask for advice from the physician’s perspective for decision-making. I would also have a face-to-face discussion with the legal representatives (besides family conference), in order to better understand the family situation (including social situation). It is not uncommon that the legal representatives disclose something new and important for the decision-making process during the discussion, which might not be available during the family conference.

Very often the family might request a trial of intensive care for several days to see if there is any response or improvement to the treatment. This is usually very helpful, not only to the family's decision-making, but also for the relief of guilt for not treating the sick patients, especially their parents. Moreover, daily communication about any progress or deterioration is important. LST will surely be continued if there are significant divergent opinions within the family, especially when the patient’s wishes are not available. However, from my personal experience, the advice of WH of LST if there is no response to intensive care therapy is often welcome and well accepted by the family, especially in cases of divergent opinions. In these cases, I probably will ask palliative care experts to join the family conference, as well as the decision-making process. Palliative care is an emerging field in China, and I believe that most families, and even some healthcare workers, do not fully understand the concept. However, my personal experience working with the palliative care team convinced me that they are in a better situation to understand the family wishes, to explain what will happen if the family/patient decline further aggressive treatment.

In China, we do not have any legal documents with regard to WH/WD LST. In addition, withdrawal of LST is seldom practised, as both the family and the healthcare workers will face the guilty feelings of accelerating the death process. As a result, WH LST is more common, although it might take days or even weeks before the patient ultimately dies.

### England

This is an 87-year-old with multiple co-morbidities. However, she is living independently with mild frailty and importantly has a potentially reversible condition. She appropriately received non-invasive ventilation in the emergency department, however this failed due to her confusion. This confusion precludes a conversation with her about her understanding of what intensive care entails and what her wishes are.

Intensivists may differ in their opinion as to whether this patient should be admitted to the intensive care [36]. However, I would have had a conversation with her family to determine whether the baseline function is really as described. I would additionally ask what an acceptable QoL would be for her (if known) and explain the potential benefits and burdens of intensive care treatment. I would explain given her underlying medical conditions that her prognosis is guarded and there is uncertainty about the expected clinical benefit of treatment in the intensive care. However, I would admit this lady for a trial of intensive care for ventilator support including invasive ventilation and low-dose vasopressors and inotropes. I would be clear from the outset that if she deteriorated and developed worsening or additional organ failure such as renal impairment, this would be evidence that she is not benefiting from intensive care. At this point after further discussion with the family, we would switch our focus of care to symptom control and palliation as the burdens of treatment now outweigh the expected benefits. Therefore, in this scenario the patient would not receive renal replacement therapy on day 2. I would ensure that there was consensus among the multidisciplinary team including another intensivist and that I have a good dialogue with the family throughout. If there were differing opinions, I would ask for a further opinion from one of my colleagues. It is not usual practice for a geriatrician to be involved in WH or WD of LST, unless they were the referring team. However, palliative care opinions are frequently sought once a decision to limit LST has been made.

The UK's General Medical Council guidelines state that if a patient lacks capacity (as is extremely common among ICU admissions) and there are no legally binding advanced directives or legal authority to make a decision on behalf of the patient, it is the treating physician's responsibility to make decisions in the patient's best interest (Table 1) [37]. Consultation with others close to the patient including family/carers and members of the healthcare team should be made before reaching a decision. In England and Wales if there is no close relative or legal proxy to represent the patient, an independent mental capacity advocate (IMCA) is required by the Mental capacity Act 2005 [38]. The IMCA can contribute to the decision, but cannot make a decision on behalf of the patient.

When making decisions about potentially life-prolonging treatments the doctor must start with a presumption in favour of prolonging life and not hastening death. However the GMC guidance states: 'there is no absolute obligation to prolong life irrespective of the consequences for the patient, and irrespective of the patient's view'. The GMC requires the clinician to weigh up the proposed benefits, burdens and risks of treatment before coming to a conclusion about the overall benefit of ongoing treatment for the patient (Table 1).

### Hong Kong SAR, China

The ageing population in Hong Kong has resulted in very old patients being increasingly referred for ICU admission. Admission triage is common in Hong Kong, with up to 15% of all admissions being declined on the basis that other patients with a greater chance of benefit are offered priority [39]. In this setting, a decision whether to admit this patient would be a carefully considered one. While the patient is very old, she has no chronic cognitive impairment, her cardiovascular function appears reasonable (although it would be useful to have an accurate indication of her pre-presentation effort tolerance), and her clinical frailty scale score is not prohibitively poor. The acute presentation of an apparent exacerbation of COPD complicated by VAP and circulatory shock, is potentially reversible with appropriate aggressive antibiotics and LST. Nevertheless, given her relatively marginal pre-morbid state, and early stage of her acute illness, a substantial degree of prognostic uncertainty exists. At most times of the year (perhaps not at times of high-pressure for ICU beds) this patient would have been admitted to the ICU. Nevertheless, a patient like this would always fall close to the conditions that could trigger a resource driven triage decision to decline admission. To better deal with the prognostic uncertainty, and offer the patient a chance of recovery without making a commitment to prolonged use of the limited resources should the patient fail to respond to ICU care positively, I would propose the institution of a time-limited trial, that would be discussed and agreed with her (if practical) and her family prior to the admission. It should be noted that many clinicians in Hong Kong would not opt for a time-limited trial, and therefore I will address the management of the patient’s progression in both contexts.

This patient did not respond positively to therapy, but in fact developed shock, followed by progressive multiple-organ failure. Generally, the time limit of a time-limited trial would have been set at 3–5 days in a case such as this, and goals for success and continuation of LST stated as resolving organ function, or signs of improving infection. In this case, these goals were not met, and the family would be sensitively informed that LST would be withdrawn, as previously agreed. Naturally, daily family update conferences would have preceded this point, and generally the decision to limit LST is well accepted by all parties, and the patient is allowed to die comfortably.

Conflict between colleagues is rare in this setting, but family insistence on continuation does occasionally occur, despite previous agreements. In this setting of divergent opinions, I would allow for some more time for discussions to reach consensus, and to clarify the patient’s best interest position, before carefully considering enforcing the limitation of LST on the basis of either the patient’s best interest, or the need to limit use of the resources available to ICU, so that they can be re-directed to patients more likely to benefit.

In the absence of a time-limited trial, the trigger for WH or WD of LST would be based on the patient’s clinical progression, and the increasing certainty that a meaningfully positive return to health was unlikely. This decision is made to serve the best-interests of the patient, and ensure a pain-free and dignified death without the provision of prolonged, burdensome and non-beneficial LST. The deterioration over the first days despite appropriate therapy, i.e. the development of shock with high vasopressor requirements and the onset of AKI, would generally result in discussion with the family regarding the poor prognosis. At the same time, it is necessary to establish if the wishes and opinions of the patient are known, or how the family believes the patient may wish to proceed. The clinical prognosis plus the latter information informs the treatment about how to determine the patient’s best interest. Once consensus between the treating team and the family is reached that the burdens of ongoing LST are no longer justified by the small likelihood of a good outcome, LST may be withdrawn. Should any prognostic uncertainty present on day 2 or 3 lead to reluctance to limit LST, by day 6, the occurrence of an embolic stroke would have resulted in greater certainty of the poor prognosis, and limitation of LST would on almost all occasions occur by this stage.

Shared decision-making is the generally accepted model for EoL care in Hong Kong. Although there is no legislation regarding EoL care, guidance issued by professional bodies (Table 1) is widely accepted [40, 41]. In daily practice, the trigger for EoL discussions and making of EoL decisions is by senior intensivists. Referrals, or support from palliative care teams or geriatricians are rare.

### Israel

The decision to admit this patient to the ICU would depend on several factors. Firstly, the potential for reversibility—would her clinical picture improve with prompt intensive management? What is the potential to bring the patient back to the previous status of health and function or, at least, to a reasonable level? Secondly, any preferences expressed by the patient regarding invasive treatments should be taken into consideration—in this case, there did not seem to be any clear directives preventing treatments such as ventilation, RRT or vasopressors. The third factor would be whether the patient could be admitted to a high care area and not an ICU. In Israel, patients are sometimes ventilated outside of the ICU in 'intermediate' or 'high care units'. These units are equipped with monitors and ventilators and staffed with 1 nurse for 4 beds. In this case, the complexity of the situation and the multi-organ failure would necessitate admission to an ICU.

In the ICU, the patient should be given a trial of full intensive care and clinical response, improvement, deterioration or complications should be assessed and re-assessed. Family discussion should be performed regularly aiming to update on treatment goals, clinical progression and planned interventions as well as support realistic expectations. At these timely clinical assessments and discussions, uncertainty may be reduced allowing for decision-making, whether to continue current treatment, escalate treatment or reduce organ support.

It is essential to allow families the time to internalise the information provided, ask questions and consult with other stakeholders, such as the primary healthcare provider, second opinions from other specialists, other family members, religious supporters and others. Family members should convey to the ICU team as much as they know about the patient's wishes. The best interest of the patient is not always clear to the ICU team or the family—What is 'suffering'? Is it prolonged in ICU? Would the patient agree to the treatment if able to communicate? What are the possible endpoints and how will they affect the well-being of the patient and burden for the caregivers? These dilemmas may sometimes be complicated by religious, legal, social and ethical issues, which may influence the decision-making even further. What is important for the ICU team is to be as clear and transparent as possible, to provide treatment options including treatment escalation but also de-escalation and allow time for changes in preferences. This patient's progression into further organ failure and the development of complications suggest a reduced chance of returning to baseline and having a reasonable QoL. The patient may remain ventilated for a prolonged period of time, dialysis dependent and bed-ridden. As these complications and setbacks develop, the ICU team and the family have the time to adjust expectations regarding ICU outcome and long-term prognosis. Geriatric assessment is useful for selecting suitable candidates for being resilient to the stress of intensive care on one hand and rehabilitation post-ICU on the other hand. A multidisciplinary team, including physiotherapists, geriatric nurse practitioners and dieticians, should evaluate the patient's potential for rehabilitation after ICU when relevant.

The Dying Patient Act [42] was passed in 2005 to define the legal framework for treating patients at the end of their life (Table 1). The law's baseline assumption is that most people want to live, however most would not want to suffer and/or be attached to artificial life support for a prolonged period. The law requires that the leading healthcare provider assesses the medical condition of the patient, his/her wishes (if known) and the response to treatment. This physician will be in charge of making a shared treatment plan with the patient, family and other stakeholders. Legal guardians or proxies can be appointed by the court. They can be involved in the shared decision-making process and give consent to interventions. 'Do not Resuscitate' orders do not require the consent of a legally appointed proxy, but usually require the agreement of family members.

If the patient in this case will not substantially improve despite maximal therapy, decisions will be made to limit further escalation of treatment and intermittent therapies, such as RTT or antibiotics. However, mechanical ventilation will continue, as it is a continuous form of life support. Palliative care will be provided, together with fluids and nutrition. In some cases, if the patient stabilises, she may be transferred to a step-down unit or a chronic ventilation facility.

### Libya

The decision to admit this patient to the ICU would have been influenced by the patient's overall health status prior to the current illness, including the severity of co-morbidities. The patient had an NSTEMI and is at risk for pulmonary hypertension due to COPD. This would need further investigation, e.g. echocardiography, to determine if the patient may tolerate aggressive treatment in the ICU. The current complaint of confusion is another essential factor to consider for admission to the ICU. Additionally, her level of functional independence prior to her current illness, the severity of her frailty, and her overall life expectancy would have been crucial factors in determining her admission to ICU. The patient was independent. However, new mobility issues due to hip osteoarthritis might have been considered, particularly if it significantly impaired her ability to carry out daily activities.

Another important aspect of consideration is her social support system, including the availability of family or caregivers who could participate in decision-making and provide care after discharge. If she lived alone without any support system, the likelihood of her returning to independent living after a severe illness might be lower. I would also have to consider the local situation. During the COVID-19 pandemic and some surges in winter or due to military conflicts in Libya, the demand for ICU admissions might be higher than available resources and the decision to admit this patient might be influenced by balancing the patient's benefit with local resources.

In cases of prognostic uncertainty, regular multidisciplinary team meetings involving physicians, nurses, medical technicians, and other relevant healthcare professionals can be organised to discuss the patient's progress, reassess prognosis, and modify the treatment plan if necessary. The ultimate responsibility would usually be with the ICU consultant in charge in the hospital.

If conscious, continuous communication with the patient and her family is crucial. They should be updated regularly about the patient's condition and prognosis, and the values, goals and preferences should be considered in decision-making. The family, notably the spouse or children, is usually involved in the early decision-making phase. If disagreements arise between healthcare providers and the family, it can be helpful to involve a neutral third party, such as the Medical Affairs Authority or another senior consultant, to facilitate discussion and help resolve the conflict. In Libya, the care of old patients is usually within the internal medicine department. Geriatric medicine and palliative care are still developing as specialties. Therefore, we usually seek advice from internists with experience in these domains.

Patients with capacity can forego any medical intervention, some patients clearly state this during admission. In some cases, physicians decide to withhold interventions after consultation with family members and explaining that active treatment may not be in the patient's best interests. This is usually influenced by the family's understanding and the local situation. Many physicians refuse to admit patients with a poor prognosis to the ICU to avoid conflicts.

Limiting LST is a controversial topic in Libya and most Middle Eastern and North African countries with similar cultural and religious belief systems. Since there are no formal guidelines or ethics recommendations for WH and WD of LST, decision-making reflects the values of individual stakeholders and differs from place to place, unit to unit, and physician to physician. There is a need for formal guidance in this domain that explains the objectives in detail and determines when and how WH and WD of LST should be done. The debate arose during the COVID-19 pandemic, when many hospitals had insufficient ICU resources.

### Norway

Although this patient had a decent QoL until 2 days prior to hospital admission, she has recently developed mobility problems and appears to be on a path to an increased level of frailty. She arrived at the hospital with three-organ failure (respiratory, cardiac, delirium) and failed the initial treatment with NIV. In my hospital, a respiratory physician would then assess the patient and decide about another trial of NIV in a respiratory intermediate care unit with support by cardiology in this case. Such a treatment trial could also provide new information for prognostication, such as remission or progression of organ failures. Importantly, severe AKI requiring RRT was documented on day 2. I would assume that this patient had some deranged renal markers already on admission to hospital. Moreover, right ventricular failure complicates positive pressure ventilation. Considering all this information, most intensivists in Norway would consider any further escalation of treatment as futile and would not admit this patient to the ICU. In Norwegian hospitals without intermediate care units, mostly in smaller hospitals, this patient might still be admitted to the ICU even without further escalation of therapy. The ICUs there are often a mixture of ICU beds, intermediate and recovery beds. After discussion among the involved healthcare professionals to reach consensus about WH of LST, the family would be summoned to communicate a decision not to intubate or provide additional organ support, such as RRT, and start palliative care. Geriatricians are only rarely available for consultation in such scenarios, but we frequently have attendance of a hospital priest.

The recommendations by the Norwegian guidelines on limitations of life-prolonging treatment relevant to this case are listed in Table 1 [43]. The decision to limit LST is made by the senior intensivist after discussion and agreement within the multidisciplinary team assigned to the patient. When possible, information about the patient's wishes must be obtained from the next-of-kin. Although neither patients nor families can demand a treatment that is considered futile, they can ask for a second opinion, usually from specialists in a different hospital. However, this is hardly feasible in an acutely life-threatening situation.

Making a judgement about futility is always challenging due to the involved uncertainty. In my opinion, there is only a small degree of prognostic uncertainty in this case, in particular from day 2. Most multi-morbid patients at this age who develop multi-organ failure within 48 h will not survive ICU. This patient probably has a SOFA score ≥ 12. To admit all similar patients to the ICU for a treatment trial to eventually have a small percentage of survivors, which probably will die shortly after discharge, is not a good way to use scarce intensive care resources in Norway. The number of ICU beds per capita is small (≈ 5 per 10^5^ inhabitants). Thus, intensivists must use resources in the most efficient way. This is reflected by the median length of stay (LOS) in the ICU for non-survivors [44] which is 1.9 days in Norway with the shortest length of stay documented in patients 80 years or older.

### Poland

This scenario represents a typical everyday dilemma about what is an appropriate admission to the ICU in terms of patients’ prognosis. It is not only the question of ICU survival, but rather the combination of this patient's likelihood to survive with a decent level of functioning and his/her own expectations concerning QoL and the burden of treatment [45].

In this scenario, an elderly women in her late 80s is brought to the hospital with signs of circulatory shock and multi-organ failure that occur on top of chronic morbidities often present with ageing. Although she lives independently, there are some mobilisation issues, and she is borderline frail (CFS 4). She is confused, and probably not capable of discussing her wishes concerning the treatment options. She fails to respond to the less invasive treatment, i.e. NIV (which in my opinion was a good choice as an initial treatment). I am quite positive, that in Poland this patient would be admitted to the ICU, if resources were available (beds, no restrictions on admissions as during the pandemic). The main reason for this decision would be the acuity of the disease, possible reversibility and relatively low frailty, which in my opinion, is the single best predictor for the outcome. I would be more hesitant to admit this patient for invasive treatment though, if it was just another exacerbation of her COPD, which deteriorated over time.

There are criteria developed by the Polish Society of Anesthesiology and Intensive Care that help decide which patient may benefit from treatment in intensive care, and whom to prioritise in terms of admission (based on the likelihood of successful treatment [46] (Table 1). Moreover, there are two position papers by medical societies that deal with preventing futile therapy which are helpful for borderline cases [47, 48]. This publication broadly describes both the clinical background of decision-making and the legal framework for preventing futile therapies. As with all guidelines, however, they should only serve as general guidance, and the decision for an individual patient should always be personalised.

With our patient admitted to the ICU I would opt for a time-limited trial of intensive care and reassess the clinical status frequently. We would try to discuss the state of the critical condition and the treatment with the patients’ relatives every day. Of note, according to Polish law, the family is not able to make decisions for the patient. There is no institution of surrogate decision-makers in Poland. Thus, the aim of these discussions is to learn more about the patient’s way of life and wishes, which helps to establish what kind of treatment could be in the best of interest for her.

From my point of view, the breaking point in this scenario is the occurrence of the embolic stroke and with subsequent hemiparesis on day 6. At this point, we would implement a ceiling of care. A decision to not perform CPR would have been taken earlier. This decision would often be made after consulting with other medical specialties, in this case most likely with neurology. Consultations with colleagues in geriatric or palliative care are not common in these situations.

After these decisions, we would discuss EoL care with the patient's family, focusing on what we can do for the patient, e.g. providing comfort, feeding, nursing, and what therapies we would withhold (e.g. new antibiotics, parenteral nutrition). In this situation, we would probably withdraw some of the interventional therapies already in place, such as RRT. We would seek the agreement of such decision with the family, who by that time, in most cases, would be prepared for the inevitable. In case of not reaching an agreement about the treatment plan, we may postpone the decision for a day or two, to have more time for discussions. However, in case of no agreement after that additional time, we would continue with moving towards palliative care as planned. I assume that in most ICUs in Poland a decision of withdrawing from mechanical ventilation would not be made, as the majority of the ICU staff would have ethical problems with that. If the patients’ condition stabilised in the following days, most probably a decision of tracheostomy would be taken and the patient transferred to a chronic ventilation facility.

## Discussion

Despite the known variability of decisions to limit LST [8, 49–52], this survey of expert opinions revealed some degree of consensus in important areas across diverse cultures. Firstly, the 87-year-old patient in the vignette scenario would be admitted to the ICU or an intermediate care unit based on the potential reversibility of the acute illness and the absence of co-morbidities deemed severe enough to interfere with critical care and subsequent recovery. However, admissions to the ICU might be affected by resource constraints in some countries. Secondly, due to the initial uncertainty about outcome, a treatment trial (with or without limitations on time) is considered desirable to obtain more prognostic information that supports discussions with family and the decision-making about LST (Table 2). Finally, the lack of response to intensive care as well as the occurrence of complications affecting survival and functional outcome would trigger a change of treatment goals from curative to comfort care, considered in the best interest of this patient.

Variations between countries surfaced regarding the specific triggers and ways to limit LST and, especially, the role of families or surrogates in the decision-making (Tables 1, 2). Differences become consequential when contemplating withholding or withdrawing invasive ventilation vs continuation of that therapy in long-term care facilities. Conflicts should be anticipated in situations where stakeholders have different belief systems, such as patients and families or healthcare professionals with different cultural backgrounds. Importantly, laws or guidelines for decisions about limiting LST are now available in most of the surveyed countries, several of those were only recently issued or updated. Some guidelines explicitly underline the importance of acknowledging cultural differences. This can be considered as an important achievement when considering the controversies in the past [53–55].

There still are important gaps and unknowns in the decision-making about limiting LST, mainly related to persistent uncertainty about survival in the ICU and its benefit for the individual patient [56]. Alternatives to ICU, i.e. intermediate care units, should be discussed early. In fact, some data suggest that admission to geriatric intermediate care units may lead to better outcomes in old patients [57]. Regardless of additional specialist input, e.g. by geriatricians to evaluate the patient's potential for functional recovery, there will always be disparate interpretations of statistical data which interfere with obtaining consensus between healthcare professionals and families about treatment goals [58]. Importantly, guidelines in some countries emphasise the probabilistic nature of prognostication. However, it is left to the discretion of intensivists to translate statistical data into decisions for the individual patient [4]. Although dealing with uncertainty and complexity is considered part of their skills set [59], providing institutional support for difficult decisions about LST, e.g. through ethics consultations, could remove part of the pressure from individual decision-makers and further increase the quality of care for both patients and families. Some cases though, may eventually require decision-making outside the healthcare system [15, 60].

Why do problems remain in this field? Firstly, challenges in decision-making for very old patients have grown in quantity and quality. In parallel to demographic ageing, there has been enormous progress in treating previously fatal conditions, such as metastatic cancer. This led to an increased influx of complex patients on the one hand and persistent enthusiasm about advanced organ support technologies on the other hand. The physical, mental and social sequelae of these interventions for very old patients and their caregivers have not yet been fully evaluated. There has not been a public discussion about the human and financial costs as well as the benefits of advanced technologies for LST and their compatibility with cultural values and societal priorities. Secondly, decision-making in the ICU mainly draws conclusions from clinical trials which were mostly focused on single interventions and did not take the burden of therapy or individual views on QoL into account. Moreover, the challenges caused by heterogeneous multi-morbidity [61] in the context of geriatric conditions were not integrated into past ICU trials in appropriate ways [62]. In numerous reports, information about limitations of LST is incomplete or absent [63]. The scarcity of comprehensive data on ICU outcome results in speculative treatment advice which frequently lacks a holistic perspective that would be particularly beneficial for very old patients. Thirdly, treatment trials in ICU to personalise decisions about LST, even when limited in time, are expensive and may further enhance ethical controversies [64]. Moreover, it still remains to be elucidated how to determine the appropriate duration of these trials [65, 66].

What needs to be done? Table 3 lists our recommendations on how to tackle some of the above issues. Most importantly, prognostic uncertainty should be acknowledged and specified in terms of survival and achievable functional outcome. Combined with an understanding of the patient's values and preferences, this approach paves the way to shared decision-making with patients, families and caregivers. Establishing an organisational framework to seek advice from other specialists, notably geriatricians, and ethics ward rounds may increase the quality of care in and after ICU and prevent burnout of healthcare professionals. Special attention should be focused on cultural differences and conflicts, especially in patients from minority groups which may have different values and expectations. There clearly is a need for more collaborative research and training in this field.

**Table 3:** Recommendations for decision-making about limiting LST in very old patients

Problem	Recommendations
Decision-making under uncertainty	Specify uncertainty (survival, functional outcome), explore potential to quantify uncertainty, establish multidisciplinary meetings and ethics ward rounds
Uncertainty about short-term outcome	Develop framework for (time-limited) ICU trial [67]
Uncertainty about potential for rehabilitation and long-term functional outcome	Consultations with geriatricians, treatment trial in rehabilitation according to the patient's phenotype [67–69]
Uncertainty about burden of therapy and benefit of outcome for the individual patient	Communicate with next-of-kin, caregivers and primary care teams to elicit patient's values and preferences [70], monitor patient's comfort
Knowledge gaps regarding survival and functional outcome in very old patients, absence of specific guidelines	Foster research and training based on international (multi-cultural) collaborations, seek advice from experts outside intensive care [45]
Variations in values and preferences among patients and healthcare professionals	Develop sensitivity to cultural differences, seek mediation and legal advice in case of divergent opinions, support advanced care planning

This article on decision-making in very old patients has a number of limitations. Firstly, we have selected specialists in intensive care according to their long-term interest in this field. Therefore, the recommendations depicted above may not be representative for the average standard of care in these countries. Moreover, the choice of countries depended on the visibility of these experts. Their number was determined by the practicalities to integrate very detailed information for each country into a single article. Secondly, opinions about limiting LST are known to vary even between intensivists in the same ICU [71]. Although our experts are working in teaching and leadership positions, a more junior generation of intensivists may develop divergent opinions. Thirdly, it is practically impossible to anticipate all scenarios which may occur in EoL situations, especially in multi-cultural settings. We, thus, kindly ask the reader to consider our statements as a set of general recommendations and stay alert to the details in specific cases.

## Conclusions

This article shows similarities and differences in the decision-making for LST in very old ICU patients and recommends strategies to deal with prognostic uncertainty. Conflicts should be anticipated in situations where stakeholders have different beliefs and expectations. There is a need for more collaborative research and training in this field.

## Data Availability

Not applicable.

## Abbreviations

AKI: Acute kidney injury
CFS: Clinical frailty scale
COPD: Chronic obstructive pulmonary disease
ICU: Intensive care unit
LST: Life-sustaining treatment
NIV: Non-invasive ventilation
QoL: Quality of life
RRT: Renal replacement therapy
VAP: Ventilator associated pneumonia
WD: Withdrawing
WH: Withholding
